# The Chromatin Regulator CHD8 Is a Context-Dependent Mediator of Cell Survival in Murine Hematopoietic Malignancies

**DOI:** 10.1371/journal.pone.0143275

**Published:** 2015-11-20

**Authors:** Jennifer R. Shingleton, Michael T. Hemann

**Affiliations:** Koch Institute for Integrated Cancer Research at MIT, Cambridge, Massachusetts, United States of America; Queen's University Belfast, UNITED KINGDOM

## Abstract

Aberrant chromatin regulation is a frequent driver of leukemogenesis. Mutations in chromatin regulators often result in more stem-like cells that seed a bulk leukemic population. Inhibitors targeting these proteins represent an emerging class of therapeutics, and identifying further chromatin regulators that promote disease progression may result in additional drug targets. We identified the chromatin-modifying protein CHD8 as necessary for cell survival in a mouse model of BCR-Abl+ B-cell acute lymphoblastic leukemia. This disease has a poor prognosis despite treatment with kinase inhibitors targeting BCR-Abl. Although implicated as a risk factor in autism spectrum disorder and a tumor suppressor in prostate and lung cancer, the mechanism of CHD8’s activity is still unclear and has never been studied in the context of hematopoietic malignancies. Here we demonstrate that depletion of CHD8 in B-ALL cells leads to cell death. While multiple B cell malignancies were dependent on CHD8 expression for survival, T cell malignancies displayed milder phenotypes upon CHD8 knockdown. In addition, ectopic expression of the Notch1 intracellular domain in a T cell malignancy partially alleviated the detrimental effect of CHD8 depletion. Our results demonstrate that CHD8 has a context-dependent role in cell survival, and its inhibition may be an effective treatment for B lymphoid malignancies.

## Introduction

Philadelphia-chromosome positive acute lymphoblastic leukemia (Ph+ B-ALL) accounts for approximately 20% of adult cases of leukemia in the United States [1]. This disease has a poor prognosis despite the development of multiple inhibitors targeting the BCR-Abl fusion tyrosine kinase that drives this disease. Patients initially respond well to tyrosine kinase inhibitors (TKIs) but quickly relapse, usually acquiring resistance due to mutations in the *Abl* kinase domain that prevent TKI binding, upregulation of drug efflux pumps, or activation of alternative signaling pathways such as SRC family kinases [2–4]. Our group previously performed a large-scale RNAi screen in a mouse model of this disease to identify factors that promote cell survival in Ph+ B-ALL and could serve as novel drug targets [5]. This model expresses a human *BCR-Abl* transgene and a disrupted *p19* ^*ARF*^ locus, closely recapitulating the genetics of the human disease as approximately 50% of Ph+ B-ALL patients exhibit loss-of-function of the CDKN2A/B locus that contains *p19* ^*ARF*^ [6,7]. In addition, most cells can give rise to disease in transplant experiments [6], so this model is able to represent a highly complex RNAi library *in vivo*.

Among the list of screening hits were shRNAs targeting chromatin modifiers with established roles in cancer such as *Sin3a*, underscoring the importance of epigenetic regulation in leukemia progression. Inhibitors of chromatin modifiers represent an emerging class of therapeutics that holds great potential, and recent work has suggested that inhibiting these enzymes could help circumvent acquired resistance to existing drugs [8–10]. Another chromatin modifier that arose as a candidate hit in this screen was *Chd8*. *Chd8* has been associated with autism spectrum disorder as well as cancer, but its mechanism of action is not well understood [11–17]. Several roles in transcriptional regulation and target gene sets have been proposed, but a consensus on the precise function of CHD8 has yet to emerge.

CHD8 was discovered in a yeast two-hybrid screen as a β-catenin binding partner that inhibits transcription of β-catenin target genes [18]. A proposed role of CHD8 is negative regulation of p53 and Wnt pathway activity through chromatin compaction at target genes during early embryonic development [19,20]. Other studies have demonstrated a role of CHD8 in cell cycle regulation including promoting transcription of E2F target genes involved in the G1/S transition [21,22].

While multiple groups have measured higher CHD8 expression in cancer cells than normal adult tissue [19,23], other groups have observed loss of expression in gastric and colorectal cancers and deletion in lung cancer [15,16,24]. It is intriguing that CHD8 appears to act in a pro-proliferative or pro-survival manner in most contexts but as a tumor suppressor in other malignancies, perhaps through inhibition of Wnt signaling. Further investigation is needed to determine the context in which CHD8 inhibition would be detrimental to the tumor and thus advantageous to patients. For this reason we pursued further investigation into the pro-survival function of CHD8 in BCR-Abl+ B-ALL cells.

We characterized *Chd8* as a pro-survival gene in this model of BCR-Abl+ B-ALL, confirming the RNAi screening results. Depletion of CHD8 resulted in cell death, but without a preceding cell cycle arrest. Interestingly, we found differing requirements for CHD8 expression between B and T cell malignancies. T-ALL cells expressing the intracellular domain of Notch (ICN) were less dependent on CHD8 expression, and ectopic expression of ICN in K-ras driven T-cell lymphoma cells partially rescued the dependency of these cells on CHD8 expression. We conclude that CHD8 is a context-dependent pro-survival factor, and that constitutive Notch signaling is able to compensate for CHD8 loss through mechanisms that are not yet fully understood.

## Materials and Methods

### Cell Culture

BCR-Abl+ B-ALL and Top Notch T-ALL cells were cultured in RPMI-1640 (HyClone) with 10% fetal bovine serum (FBS), 5μM β-mercaptoethanol, and 4mM L-glutamine. Eu-*myc Arf* ^*-/-*^ cells were cultured in 45% DMEM/45% IMDM (HyClone) with 10% FBS, 5μM β-mercaptoethanol, and 2mM L-glutamine. *K-ras* ^*LA2/+*^ *; p53* ^*LSL/LSL*^ T cell lymphoma cells were cultured in IMDM with 10% FBS and 10μM β-mercaptoethanol. Pre-B cells were harvested from bone marrow of a C57BL/6 mouse, stained with fluorescently-conjugated anti-B220 (BioLegend), anti-CD11b (eBioscience), and anti-IgM antibodies (eBioscience) and sorted to obtain B220^+^CD11b^-^IgM^-^ cells. Pre-B cells were cultured in 45% DMEM/45% IMDM with 10% FBS, 5μM β-mercaptoethanol, 2mM L-glutamine, recombinant murine IL-7 (1.0 ng/mL), and recombinant murine SCF (1.0 ng/mL) (Peprotech) on a feeder layer of bone marrow stromal cells.

### shRNAs and plasmids

shRNAs were designed and cloned as previously described [25]. Oligo sequences (S1 Table) were PCR-amplified with primers containing XhoI and EcoRI restriction sites (S2 Table). shRNAs were cloned into MSCV/LTRmir30-PGK-puromycin^r^-IRES-GFP (MLP) or MSCV/LTRmir30-SV40-GFP (MLS) for GFP competition assays, and TRMPVIR (TRE-dsRed-miR30/shRNA-PGK-Venus-IRES-rtTA3) for inducible shRNA studies [26]. pMIG (MSCV-IRES-GFP, Addgene 9044) and pMIG-ICN were used for rescue assays. *Duplin* was cloned using the Gibson Assembly^®^ method (New England BioLabs). mRNA was extracted from B-ALL cells using a Qiagen RNEasy kit and reverse-transcribed with the ThermoScript RT-PCR system (Life Technologies). cDNA was PCR-amplified with Phusion polymerase (New England BioLabs) with primers flanking the *Duplin* sequence and containing Gibson Assembly^®^ overhang sequences (S2 Table). The PCR product was ligated into modified pMIG (Not1 and Mfe1 sites inserted between EcoRI and XhoI restriction sites using oligos listed in S2 Table). To generate retroviruses, 293T cells were transfected with plasmids using the calcium phosphate method [27].

### CRISPR-Cas9

Single-guide RNA sequences (S3 Table) were designed and cloned into pSpCas9(BB)-2A-GFP according to the protocol in Ran *et al* [28]. tdTomato+ B-ALL cells were transfected using Lipofectamine 3000 (Life Technologies) according to the manufacturer’s protocol. Cells were sorted 24 hours later by GFP expression, either into 96-well plates as single cells, or into tubes and seeded into 96-well plates as single cells 3 days later. Clonal populations were analyzed for CHD8 expression by western, and editing of the *Chd8* gene by sequencing (PCR and sequencing primers listed in S2 Table). For growth competition assays, clonal populations were mixed with unlabeled control B-ALL cells and seeded in triplicate. Percentages of tdTomato+ cells were analyzed 2, 4, and 8 days later by flow cytometry. Clones Chd8-1 and -2 were generated with sgChd8-2, clone Chd8-4 was generated with sgChd8-3, and clones Chd8-3 and Chd8-5 were generated with sgChd8-4. Clones Ren-1 and -2 were generated with sgRen-2, and clone Ren-3 was generated with sgRen-3.

### Western blotting and qPCR

Cell pellets were generated following puromycin selection (MLP) or doxycycline treatment of sorted cells (TRMPVIR). *K-ras* ^*LA2/+*^ *; p53* ^*LSL/LSL*^ cells were sorted following transduction with MLS. Lysates were generated using RIPA buffer. Antibodies and dilutions are listed in S4 Table. mRNA for qPCR analysis was extracted using a Qiagen RNEasy kit and reverse-transcribed with MMLV-RT (New England BioLabs). qPCR primer sequences are listed in S5 Table. qPCR was performed using Fast SYBR^®^ Green Master Mix and a StepOnePlus^TM^ Real-Time PCR System (Applied Biosystems).

### Growth competition assays and survival experiments

Cells were partially infected with the indicated retroviruses and seeded in 6-well plates in triplicate. Percentages of GFP+ cells were determined by flow cytometry on days 2, 6, and 10 after infection. For *in vivo* competition assays, 2×10^6^ partially infected cells were injected into female C57BL/6 (6–8 week old) mice via the tail vein. Upon disease presentation, leukemic cells were harvested from the spleen, bone marrow, and peripheral blood and analyzed by flow cytometry to determine percentages of GFP+ cells. Propidium iodide incorporation was used to exclude dead cells. For survival experiments, cells transduced with the indicated constructs were sorted by GFP expression and approximately 20 GFP+ cells per mouse (female C57BL/6, 6–8 weeks old) were injected via the tail vein. Upon disease presentation, cells were harvested from the spleen and peripheral blood and analyzed by flow cytometry to determine percentages of GFP+ cells. All mice were sourced from Jackson Laboratories.

### Growth curves and cell cycle analysis

Cells were infected with MLP, MLP-sh*Chd8*-0, or MLP-sh*Chd8*-1 and selected with puromycin. Indicated numbers of live cells were seeded in triplicate, and total numbers of live and dead cells were counted at the indicated time points by hemocytometer and trypan blue incorporation. For cell cycle analysis, B-ALL cells were infected with TRMPVIR-shRen or TRMPVIR-sh*Chd8*-1 and sorted by Venus expression. Sorted cells were plated and treated with doxycycline (Sigma, 200 ng/mL), and samples were collected at indicated time points and fixed in ethanol. Cells were stained with propidium iodide and analyzed by flow cytometry. Cell cycle profiles were created with ModFit LT software (Verity Software).

### Statistical analysis

Student’s t tests, ANOVA, and survival analyses were performed with GraphPad Prism software.

### Ethics Statement

This study was carried out in accordance with the recommendations in the Guide for the Care and Use of Laboratory Animals of the NIH. The protocol was approved by the MIT Committee on Animal Care (Protocol #0515-044-18). All efforts were made to minimize suffering. In long-term survival experiments, animals were monitored 3 times per week for two weeks (a time-frame established by prior experiments with this disease model), then daily as animals in the control cohort developed disease. Animals were euthanized when symptoms (hunched posture, lower levels of activity) were displayed. There were no unexpected animal deaths.

## Results

### CHD8 depletion is detrimental to growth of BCR-Abl+ B-ALL cells *in vitro* and *in vivo*

**Fig 1:**
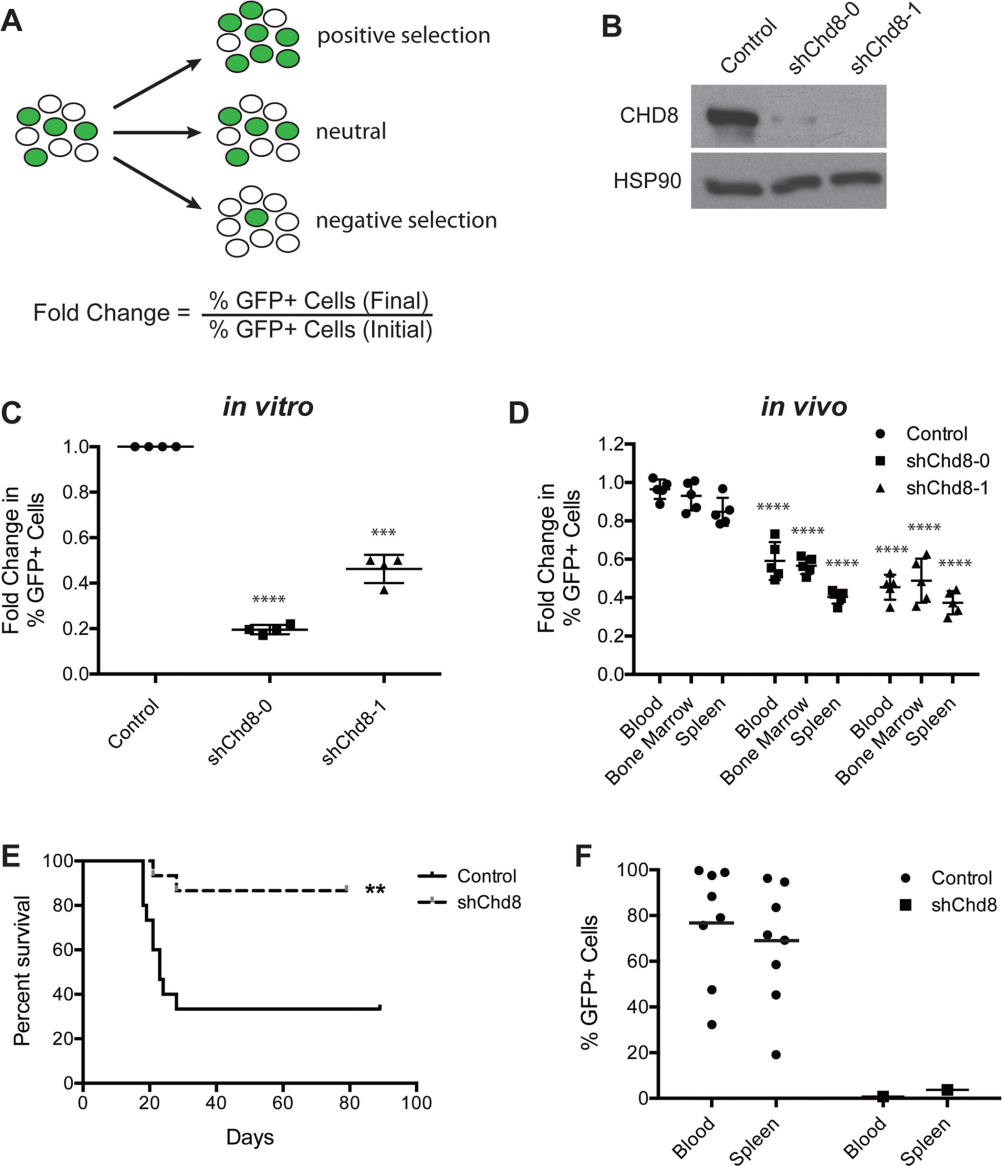
CHD8 depletion by RNAi is detrimental to B-ALL cells *in vitro* and *in vivo*. **(A)** Schematic of the GFP growth competition assay. An increase in the percentage of GFP+ cells indicates positive selection conferred by shRNA expression, while a decrease indicates negative selection. **(B)** Western blot showing decreased CHD8 expression in B-ALL cells conferred by both shRNAs (4 days following retroviral infection). **(C, D)** Results of *in vitro* **(C)** and *in vivo* **(D)** growth competition assays. Shown are averages ± SEM of four independent experiments (C) and averages ± SD of 5 mice (D). Fold changes of *in vitro* assays were calculated from day 2 to day 10 after retroviral infection and normalized to an empty vector control. Fold changes of *in vivo* assays were calculated from day 2 after retroviral infection (day of injection) to morbidity (approximately 10 days later). *In vivo* assay shRNA results were compared with empty vector control in each respective tissue. **(E)** Kaplan-Meier curve showing survival of mice injected with B-ALL cells transduced with the indicated constructs. n = 15 per cohort. **(F)** Graph showing GFP expression of cells harvested from the indicated mice upon morbidity. **P < 0.005, ***P = 0.002, ****P < 0.0001

To validate that the deleterious effect of the original shRNA targeting *Chd8* in the screen is due to knockdown of the intended target, additional constructs targeting *Chd8* were designed and tested by GFP competition assays (Fig 1A). In these assays, a population of cells is partially transduced with a retroviral vector expressing an shRNA linked to a GFP marker to allow identification of shRNA-expressing cells by flow cytometry. As with the original shRNA identified by the screen, an additional shRNA also led to a significant decrease in CHD8 expression at the protein level (Fig 1B). Consistent with an on-target effect, both constructs led to depletion of transduced B-ALL cells over time, both in *in vitro* and *in vivo* settings (Fig 1C and 1D). Depletion of sh*Chd8-*expressing leukemic cells was observed in all lymphatic tissues examined (spleen, bone marrow, and peripheral blood). These results indicate that the detrimental effects of CHD8 knockdown are cell-autonomous and independent of the tumor microenvironment. Interestingly, while sh*Chd8*-0 appears to confer its deleterious phenotype more rapidly than sh*Chd8*-1 *in vitro*, cells expressing sh*Chd8*-1 deplete to a similar extent when examined over a longer period of time (S1 Fig).

The model of BCR-Abl+ B-ALL utilized in this study is very aggressive, giving rise to terminal disease in less than two weeks following tail vein injection of 2 million cells into immunocompetent syngeneic mice. We predicted that CHD8 depletion in transplanted B-ALL cells would increase time to disease due to attenuation of cell viability and tumor growth. Cells expressing sh*Chd8*-1 or a GFP vector control were sorted to obtain pure GFP+ populations. Very low numbers of cells were transplanted by tail vein injection into each of 30 C57BL/6 mice (15 per cohort), and time to morbidity was monitored. Time to terminal disease was indeed extended significantly in mice injected with CHD8-depleted cells (Fig 1E). Disease penetrance was decreased as well, with only 2 mice in the sh*Chd8*-1 cohort succumbing to disease, compared with 10 mice in the control cohort. When mice reached morbidity, cells from the spleen and peripheral blood were collected and analyzed by flow cytometry for GFP expression. Interestingly, cells collected from mice in the control cohort were polyclonal and expressed GFP, but cells harvested from mice injected with CHD8-depleted cells were GFP-negative (Fig 1F). These results indicate that disease onset in these mice was caused by the outgrowth of a small number of GFP-negative cells contaminating the injected population.

**Fig 2:**
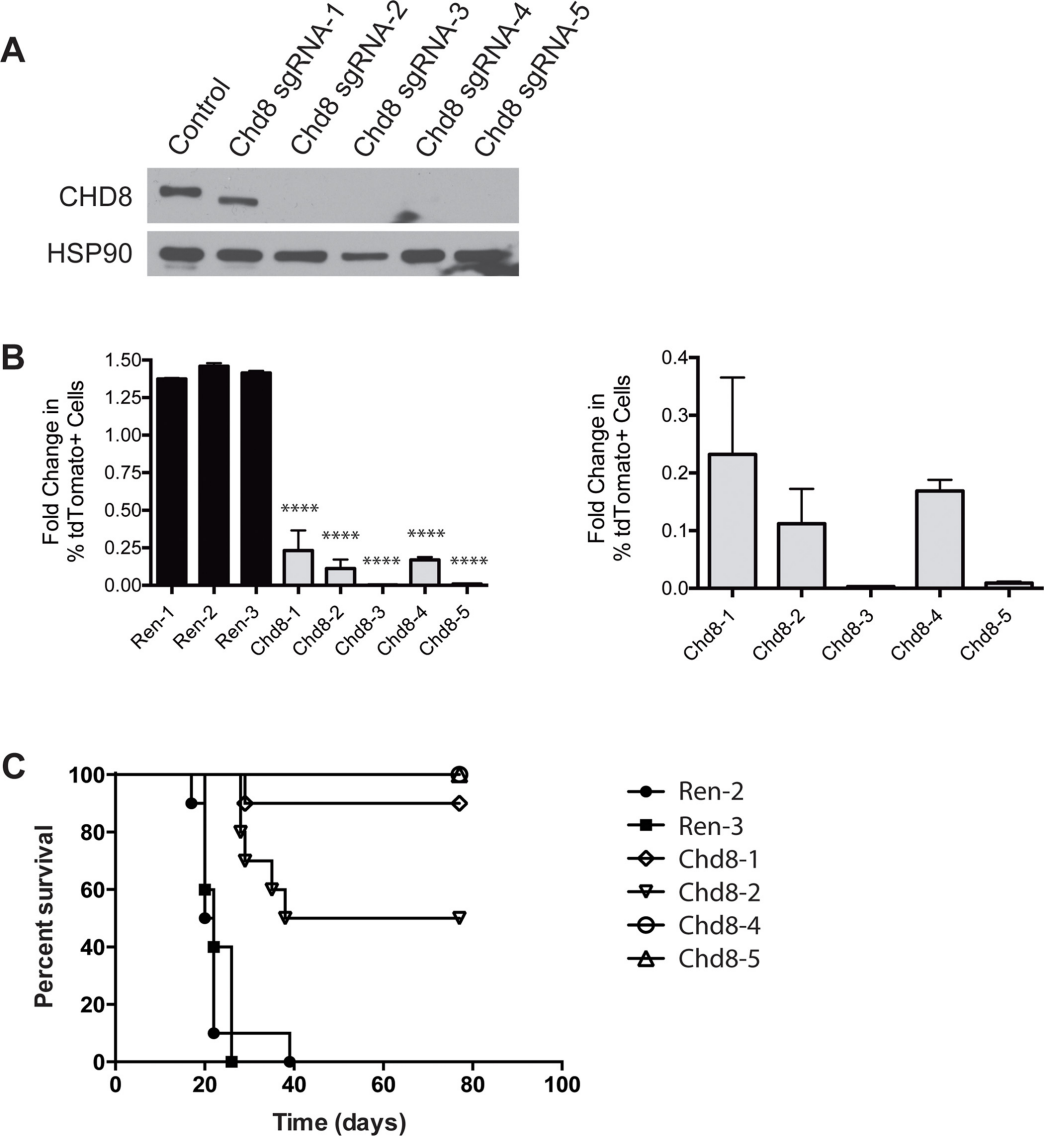
CRISPR-Cas9 editing of CHD8 is detrimental to B-ALL cells. **(A)** Western blot showing loss of CHD8 expression or expression of a truncated protein following gene editing by CRISPR-Cas9 in B-ALL cells. Expression of truncated protein likely arises from an ORF in original reading frame following a new stop codon generated by gene editing. Chd8 sgRNA-1 and -2 cell lines were generated with sgChd8-2 construct, Chd8 sgRNA-4 was generated with sgChd8-3 construct, and Chd8 sgRNA-3 and -5 were generated with sgChd8-4 construct. **(B)** (Left) Bar graph showing results of *in vitro* growth competition assays with B-ALL cells following gene editing by CRISPR-Cas9. Constructs targeting Renilla luciferase (Ren) were used as negative controls. Ren-1 and -2 cell lines were generated using sgRen-2 construct, and Ren-3 was generated using sgRen-3 construct. Shown are averages ± SD. (Right) Detailed bar graph showing results of *in vitro* growth competition assays. **(C)** Kaplan-Meier curve showing survival of mice injected with B-ALL cells transduced with the indicated constructs. n = 10 per cohort. See S6 Table for statistics. ****P < 0.0001

As additional confirmation that the detrimental effects of these shRNAs are due to depletion of CHD8, we performed gene editing using the CRISPR-Cas9 system. B-ALL cells expressing the tdTomato fluorophore were transfected with one of three single-guide sequences cloned into a CRISPR-Cas9 vector. Single cell clones were generated and tested for gene editing and loss of CHD8 protein expression (Figs 2A and S2). These cells were mixed with unlabeled control B-ALL cells to carry out *in vitro* growth competition assays. We observed depletion of tdTomato+, *Chd8*-edited B-ALL cells relative to control cells in these assays, confirming that loss of CHD8 is detrimental (Fig 2B). In contrast, B-ALL cells transduced with guide sequences targeting Renilla luciferase, which is not expressed in these cells, expressed normal levels of CHD8 (S3 Fig) did not deplete in similar growth competition assays (Fig 2B). In addition, mice were injected with low numbers of B-ALL cells grown from these CHD8-deficient or control clonal populations (10 mice per cohort). All 20 mice injected with control B-ALL cells succumbed to disease by Day 39 after injection, but only 6 out of 40 mice injected with cells deficient for CHD8 developed disease within 11 weeks of injection (Fig 2C).

### An inducible RNAi vector allows examination of gene function in a temporally controlled manner

**Fig 3:**
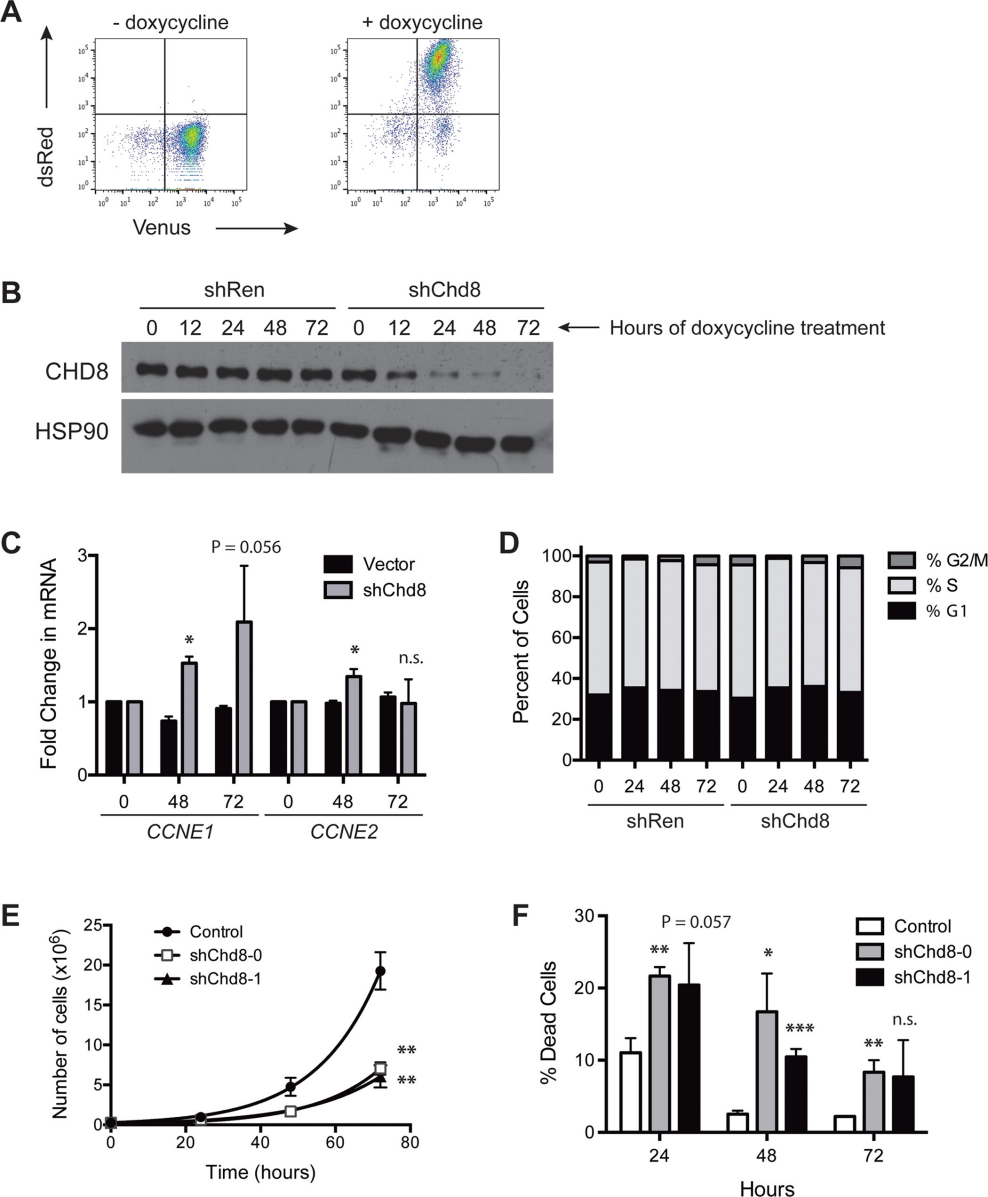
CHD8 knockdown in B-ALL cells leads to cell death without a cell cycle arrest. **(A)** Flow cytometry plots showing induction of dsRed and a linked shRNA upon treatment with doxycycline. **(B)** Western blot showing decreased CHD8 expression over time following induction of sh*Chd8* in B-ALL cells by doxycycline. **(C)** Bar graph showing increased *CCNE1* and *CCNE2* expression upon CHD8 knockdown. Shown are averages ± SD. **(D)** Bar graph showing cell cycle profiles of B-ALL cells following shRNA induction by doxycycline. **(E)** Graph showing growth rates of B-ALL cells transduced with the indicated constructs. Shown are averages ± SD. **(F)** Bar graph showing percentages of dead cells following *Chd8* knockdown. Survival of cells transduced with sh*Chd8* may be due to selection against CHD8 knockdown. Shown are averages ± SD. *P < 0.01, **P < 0.005, ***P < 0.0005

In order to study the downstream molecular effects of *Chd8* knockdown in a temporally controlled manner, an shRNA targeting *Chd8* was cloned into the doxycycline-inducible retroviral vector TRMPVIR [26]. This vector utilizes a Tet-On system to control transcription of an shRNA. A reverse Tet-transactivator (rtTA) and the Venus fluorophore are constitutively expressed, and addition of doxycycline induces expression of the shRNA as well as a dsRed marker, creating a population of Venus^+^dsRed^+^ cells (Fig 3A). When transduced into B-ALL cells, TRMPVIR-sh*Chd8*-1 leads to a marked reduction of CHD8 expression by 12 hours following treatment with doxycycline, while CHD8 levels in cells transduced with TRMPVIR expressing a Renilla luciferase shRNA (shRen) are not affected (Fig 3B).

### CHD8 depletion leads to cell death without a preceding cell cycle arrest

Others have found that CHD8 depletion leads to a decrease in expression of *CCNE2* and *TYMS* and a subsequent cell cycle arrest at the G1/S transition [21]. We examined *CCNE2* as well as *CCNE1* expression by qPCR in B-ALL cells expressing TRMPVIR-sh*Chd8*-1 or the shRen control. Surprisingly, mRNA expression levels of *CCNE1* and *CCNE2* were found to be slightly higher in CHD8-depleted cells after 48 hours of doxycycline (Fig 3C). This result seemed counter to the expected outcome in that a decrease in cyclin expression rather than an increase would be expected to be detrimental to cell cycle progression. To determine if this change in *CCNE1* and *CCNE2* levels correlated with a change in cell cycle profile, cell cycle analysis was carried out on these populations. Flow cytometry analysis revealed no significant change in the cell cycle profile upon CHD8 depletion (Fig 3D), indicating that dysregulation of *CCNE1* and *CCNE2* transcription upon CHD8 depletion does not lead to a G1/S arrest in B-ALL cells.

However, these results do not preclude the possibility that CHD8 depletion leads to a prolonged doubling time in these cells, lengthening each stage of the cell cycle rather than arresting in a particular stage. CHD8 depletion could also lead to cell death without a preceding cell cycle arrest. To test this possibility, we retrovirally infected cells with a puromycin-selectable vector expressing one of two sh*Chd8* constructs or a vector control. Following puromycin selection, 2.5×10^5^ live cells per construct were plated in triplicate, and the total numbers of live and dead cells were quantified at 24-hour intervals. Both sh*Chd8* constructs caused a decrease in the apparent growth rates of B-ALL cells and an increase in the percentages of dead cells (Fig 3E and 3F). Taken together, these results argue that the depletion of sh*Chd8*-expressing cells observed in the growth competition assays is due to cell death rather than slowed proliferation.

Notably, this death may not be due to canonical apoptosis as treatment with the pan-caspase inhibitor ZVAD-fmk simultaneously with doxycycline did not prevent CHD8 knockdown-mediated cell death an in *in vitro* growth competition assay (S5 Fig). In addition, no evidence of caspase 3 cleavage was seen by western blot at 12, 24, 48, or 72 hours after doxycycline treatment (S6 Fig).

### Ectopic expression of CHD8 N-terminal domains

**Fig 4:**
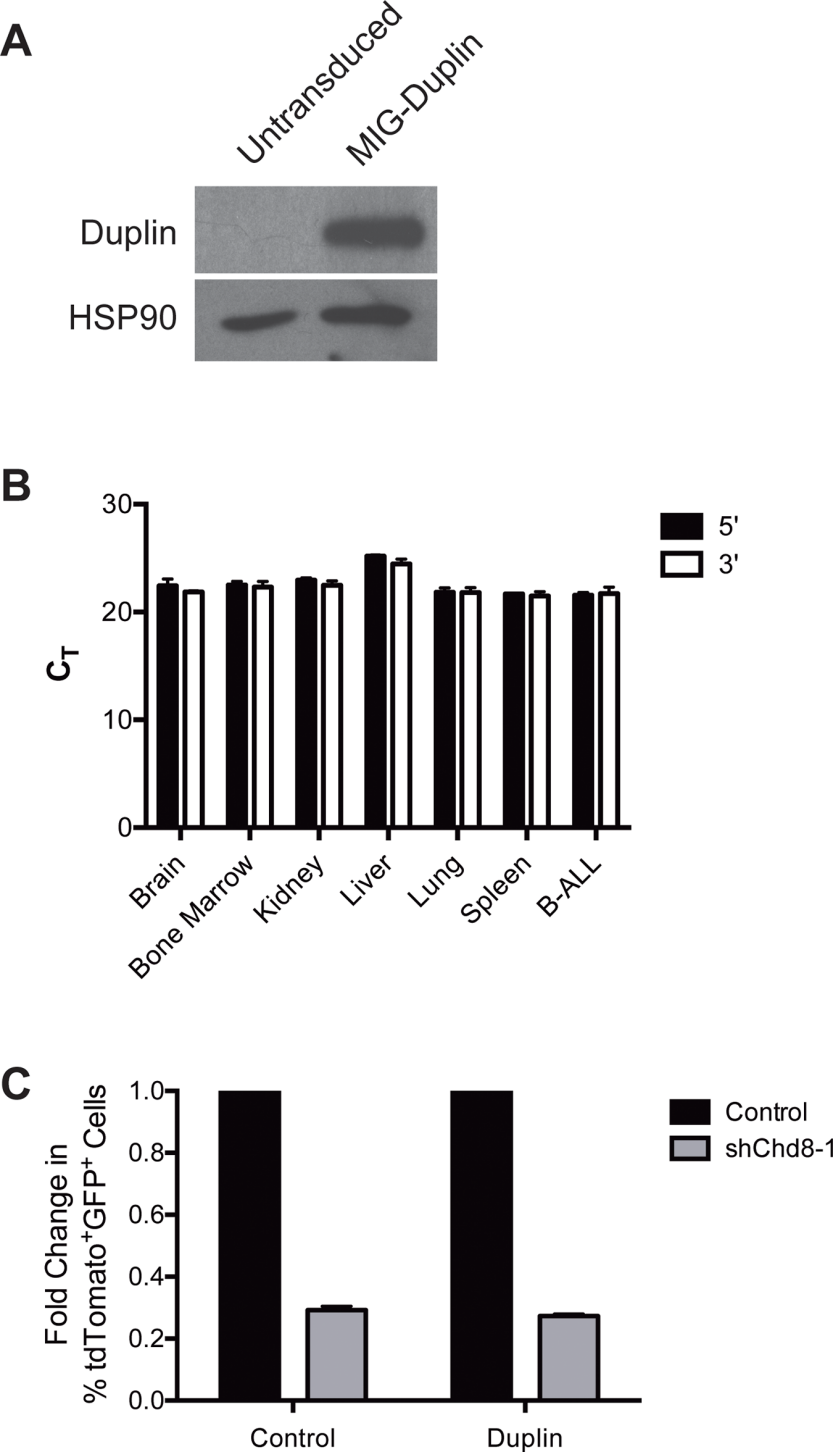
Duplin expression does not rescue shChd8 phenotype in B-ALL cells. **(A)** Western blot showing Duplin expression in B-ALL cells transduced with *MIG-Duplin*, but not untransduced cells. **(B)** Bar graph showing *Chd8* C_T_ values in the indicated mouse tissues with the indicated qPCR primer pairs. Shown are averages ± SD. **(C)** Bar graph showing depletion of sh*Chd8*-expressing B-ALL cells transduced with *MIG-Duplin* or a GFP vector control. Shown are averages normalized to vector control ± SD. Fold changes were calculated from day 2 to day 10 after second retroviral infection and normalized to MLT (tdTomato) empty vector control.

Given that a number of CHD8 domains have been shown to interact with specific binding partners including p53, β-catenin, CTCF, and methylated H3K4 [19,20,29], we reasoned that determining the domains necessary for CHD8 function in B-ALL cells might inform important pro-survival roles. Previously, others have found that overexpression of a 110 kDa truncated N-terminal isoform of murine CHD8 called Duplin was able to rescue cells from p53-mediated apoptosis as effectively as full-length CHD8 [19]. Duplin contains the first of the two chromodomains in CHD8, but not other identified functional domains [20,29]. We hypothesized that overexpression of this truncated isoform might be able to rescue the sh*Chd8* phenotype in B-ALL cells. *Duplin* cDNA was PCR-amplified from B-ALL cDNA and cloned into an MSCV-IRES-GFP (pMIG) retroviral vector. Transduction of B-ALL cells with pMIG-*Duplin* led to high expression of the protein as expected, however endogenous expression of Duplin was not observed in untransduced cells (Fig 4A). Previous studies did not measure Duplin expression in blood, so it is possible that only the full-length isoform is expressed in murine lymphoid cells. However, we were also unable to detect expression of multiple isoforms of *Chd8* by qPCR in other murine tissues including brain, liver, lung, and kidney (Fig 4B). Although this isoform may not normally be expressed, we reasoned that if the domains found in Duplin are important for CHD8 function in B-ALL cells, Duplin overexpression would still be able to prevent depletion of sh*Chd8*-expressing cells in growth competition assays. sh*Chd8*-1 was selected for use in rescue experiments as it is specific for the full-length isoform. As seen in Fig 4C, exogenous expression of Duplin was unable to prevent depletion of sh*Chd8*-expressing cells, suggesting that the domains found in Duplin are not sufficient to rescue the effects of CHD8 depletion. While a full-length cDNA control that does rescue the CHD8 knockdown phenotype would make these results more conclusive, the large size of this cDNA (7.75 kb) made it challenging to clone and express in B-ALL cells despite several attempts.

### Untransformed pre-B cells require CHD8 for survival

**Fig 5:**
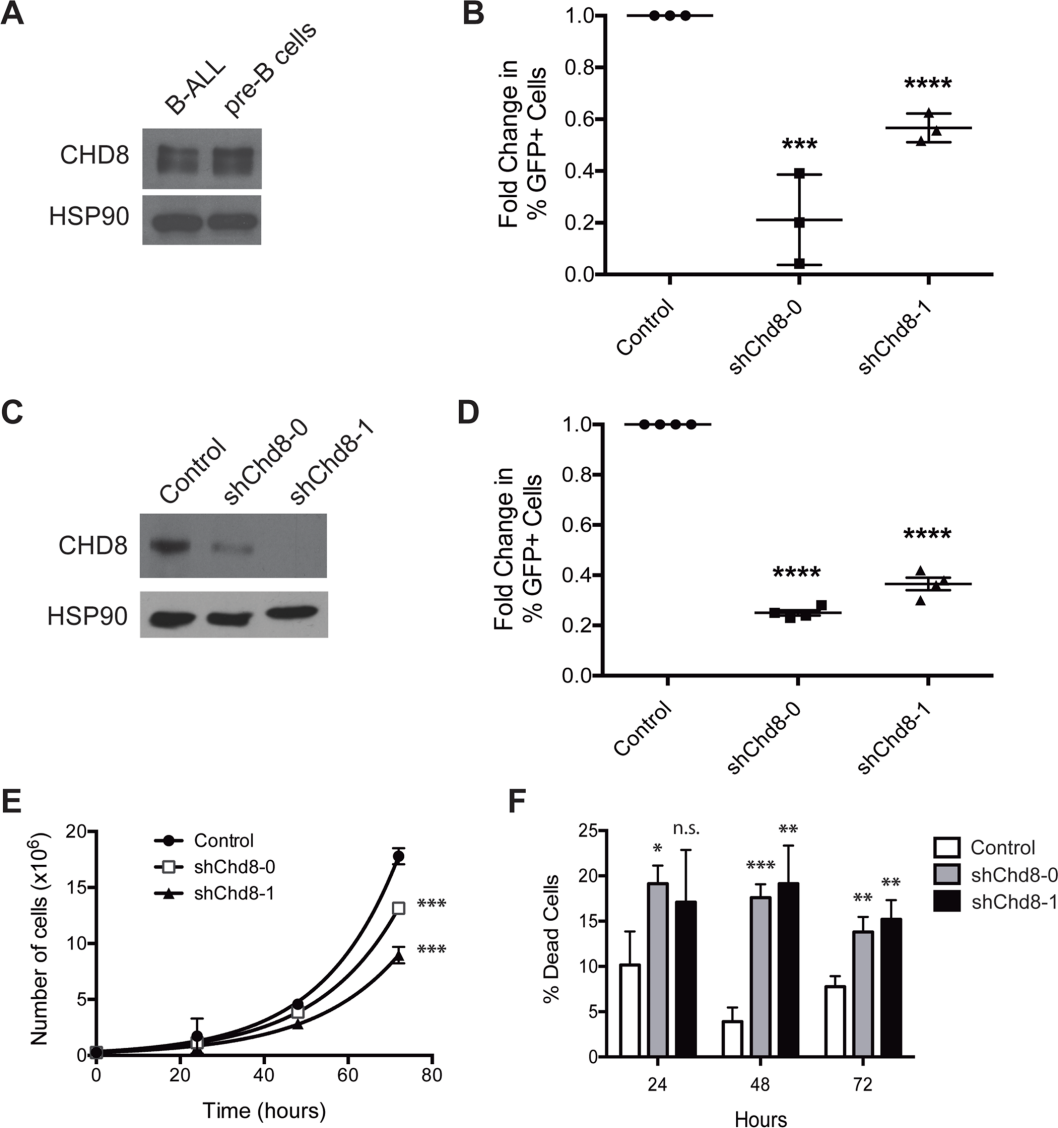
CHD8 depletion is detrimental in multiple B cell malignancies and normal pre-B cells. **(A)** Western blot showing CHD8 expression in B-ALL cells and untransformed pre-B cells. **(B)** Graph showing results of growth competition assay with pre-B cells transduced with the indicated constructs. Shown are averages ± SD. Fold changes were calculated from day 2 to day 10 after retroviral infection and normalized to an empty vector control. **(C)** Western blot showing decreased CHD8 expression following transduction of Eμ-myc *Arf* ^*-/-*^ cells with the indicated sh*Chd8* constructs. **(D)** Graph showing results of *in vitro* growth competition assays with Eμ-myc cells transduced with the indicated constructs. Shown are averages ± SEM of four independent experiments. Fold changes were calculated from day 2 to day 10 after retroviral infection and normalized to an empty vector control. **(E)** Growth curves of Eμ-myc cells transduced with the indicated constructs. Shown are averages ± SD. **(F)** Bar graph showing percentages of dead cells following *Chd8* knockdown in Eμ-myc cells. Shown are averages ± SD. *P < 0.05, **P < 0.01, ***P < 0.001, ****P < 0.0001

Previously, it had been observed that CHD8 is overexpressed in murine cancer cell lines compared to corresponding normal tissues [20]. To determine if this pattern could be observed in the B-cell lineage, bone marrow was harvested from healthy adult C57BL/6 mice, and pre-B cells (B220^+^IgM^-^CD11b^-^) were collected by flow cytometry. Unexpectedly, CHD8 expression in these untransformed pre-B cells was comparable to that seen in B-ALL cells (Fig 5A). The dependence of these cells on CHD8 expression was also tested by a growth competition assay *in vitro*. While protein expression levels in these cells may be similar, biological differences between the cell types might make untransformed pre-B cells less reliant on CHD8 for survival. However, untransformed pre-B cells expressing sh*Chd8* depleted *in vitro* to a similar extent as B-ALL cells, indicating that in the B lymphoid lineage dependence on CHD8 expression is not limited to malignant cells (Fig 5B).

### Differential requirement for CHD8 expression in hematopoietic malignancies

While CHD8 expression is required in untransformed pre-B cells, malignancies with different genetic backgrounds or within different developmental contexts may have mechanisms that allow cell survival in the absence of this protein. We reasoned that identifying a context-specific survival requirement could shed light on CHD8 function. To determine whether dependency on CHD8 extends to other hematopoietic malignancies, we first tested another cancer of the B cell lineage, a murine model of Burkitt’s lymphoma. In this model, c-*myc* is under the transcriptional control of the μ immunoglobulin heavy chain enhancer (Eμ-*myc*), mimicking the t(8;14)(q24;q32) chromosomal translocation that causes this disease in humans [30–32]. We transduced these cells with shRNAs targeting *Chd8* and confirmed decreased protein expression by western blot (Fig 5C). As with BCR-Abl+ B-ALL cells, Eμ-*myc* cells expressing these shRNAs deplete in *in vitro* growth competition assays (Fig 5D), and pure populations of shRNA-expressing cells display decreased proliferation rates and increased numbers of dead cells compared with cells expressing vector controls (Fig 5E and 5F).

**Fig 6:**
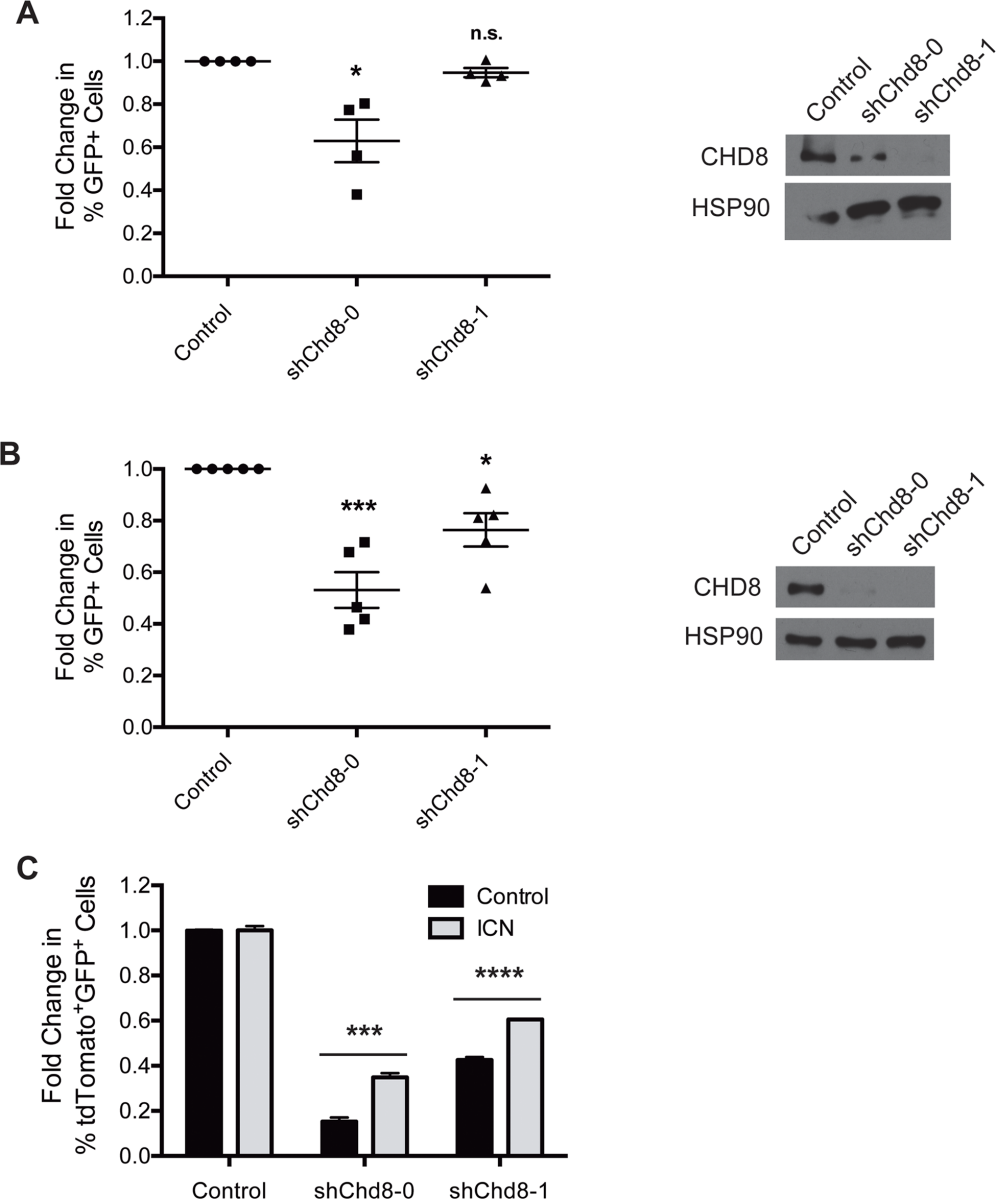
T cell malignancies exhibit different requirements for CHD8 expression that can be partially alleviated by constitutive Notch signaling. **(A)** (Left) Graph showing results of *in vitro* growth competition assays with Top Notch cells. Shown are averages ± SEM of four independent experiments. (Right) Western blot showing decreased CHD8 expression in Top Notch cells following transduction with sh*Chd8*-0 or -1. **(B)** (Left) Graph showing results of *in vitro* growth competition assays with KP lymphoma cells. Shown are averages ± SEM of five independent experiments. (Right) Western blot showing decreased CHD8 expression in KP lymphoma cells following transduction with sh*Chd8*-0 or -1. Fold changes of competition assays were calculated from day 2 to day 10 after retroviral infection and normalized to an empty vector control. **(C)** Graph showing results of ICN rescue experiment with KP lymphoma cells. Shown are averages ± SD from a representative experiment. Fold changes were calculated from day 2 to day 10 after second retroviral infection and normalized to MLT empty vector control. *P < 0.05, ***P < 0.0005, ****P < 0.0001

A recent study into vulnerabilities of drug-resistant T-ALL cells uncovered a number of epigenetic regulators that became essential upon acquisition of drug resistance [8]. CHD8 was one of several epigenetic regulators found to be preferentially required for survival by γ-secretase inhibitor (GSI)-resistant cells over syngeneic, GSI-sensitive cells. These resistant cells exhibited a lower level of Notch signaling than the sensitive population, suggesting that high Notch pathway activity may somehow compensate for CHD8 loss. In agreement with these results, we found that a T-ALL cell line that constitutively expresses the intracellular domain of Notch (“Top Notch”) [33], and thus exhibits constitutive activation of the Notch pathway, is less dependent on CHD8 expression for survival than the B cell malignancies tested. Top Notch cells expressing sh*Chd8*-1 did not deplete in *in vitro* growth competition assays (Fig 6A). While sh*Chd8*-0 caused depletion that was determined to be statistically significant, this depletion was less than that seen in B-ALL cells.

We also examined CHD8 dependency in a spontaneous T-cell lymphoma cell line derived from a *K-ras* ^*LA2/+*^ *; p53* ^*LSL/LSL*^ mouse (hereafter referred to as KP). These cells express oncogenic K-ras^G12D^ and are functionally p53-null [34,35]. KP lymphoma cells deplete upon CHD8 knockdown in growth competition assays, but to a lesser extent than B cell malignancies (Fig 6B). The distinction between CHD8 dependencies in these two T cell populations could shed light on the mechanism of CHD8 activity. To determine if constitutive Notch signaling could rescue the CHD8 knockdown phenotype, KP lymphoma cells were transduced with the Notch intracellular domain (ICN) linked to a GFP marker, or a GFP marker alone (S7 Fig) [36]. Ectopic ICN expression is lethal to B cells [37], so they could not be used for these experiments. Transduced KP cells were sorted to obtain pure GFP+ populations, then partially transduced with one of two sh*Chd8* constructs linked to a tdTomato marker, or a vector control. Cells were analyzed by flow cytometry to assess the change in percentage of tdTomato+ cells in each population. While cells expressing sh*Chd8* on the GFP+ control background depleted to a significant extent, cells expressing sh*Chd8* on the ICN-transduced background were partially rescued from depletion (Fig 6C). This result indicates that constitutive Notch signaling can attenuate the detrimental effects of CHD8 knockdown in KP lymphoma cells, possibly overriding a reduction in survival or growth signals caused by CHD8 depletion that other oncogenic signaling pathways are unable to compensate for.

## Discussion

We have demonstrated that CHD8 is necessary for survival of B lymphoid malignancies. This dependency exists in both the *in vitro* and *in vivo* settings, indicating that CHD8 functions in a cell-autonomous manner and its depletion does not alter interactions of B-ALL cells with their microenvironment. When we examined the effects of CHD8 depletion on leukemic cell proliferation, we uncovered a cell death phenotype not preceded by cell cycle arrest. Others have shown that CHD8 knockdown in cervical carcinoma cells leads to G1 arrest facilitated by downregulation of *CCNE2* and *TYMS*, genes required for transition into S phase [21]. However, when we examined cell cycle profiles of B-ALL cells upon CHD8 knockdown, we observed no difference between control and CHD8-depleted cells. It is possible that the differences between our results and those of Rodriguez-Paredes *et al*. are due to the distinct signaling contexts in the two cell types used.

Several early studies observed expression of a truncated N-terminal CHD8 isoform termed Duplin [18,19]. We found that exogenous expression of Duplin did not compensate for CHD8 knockdown, suggesting that domains unique to full-length CHD8 are crucial for its function in B-ALL cells. The second chromodomain, absent in Duplin, has been shown to enable binding to histone H3 [21], and the BRK domains at the C-terminus have been demonstrated to interact with the chromatin insulator CTCF [29]. It is curious that, unlike results of previous studies, endogenous Duplin expression was not found in B-ALL cells or in normal mouse tissues by qPCR. Bioinformatics databases currently show truncated isoforms of CHD8 only in rat, so it is likely that murine Duplin is an experimental artifact not physiologically expressed.

Previous work by our group has highlighted genes whose effect on survival is context-specific between B and T cell malignancies [5]. For example, the chromatin regulator PHF6 is required for survival of B-ALL cells, but loss-of-function is selected for in T-ALL [38]. Factors such as HES1 that promote T lineage development are often lethal when ectopically expressed in the B cell lineage [37]. We found that Top Notch T-ALL cells are significantly less dependent on CHD8 expression than the B cell malignancies tested. The results of the ICN rescue experiment indicate that constitutive Notch signaling can partially compensate for CHD8 knockdown in a T cell malignancy, consistent with results of a recent screen for chromatin regulators that are essential in GSI-resistant cells [8]. CHD8 was among the proteins found to be necessary for survival of resistant cells but not syngeneic GSI-sensitive cells that continued to exhibit high levels of ICN and a more open chromatin conformation. These results suggest that CHD8 and high Notch signaling are able to compensate for each other’s absence through mechanisms that are currently unclear. It is possible that CHD8 compensates for chromatin compaction upon Notch inhibition by promoting transcription. Alternatively, CHD8-mediated chromatin compaction could promote survival of GSI-resistant cells that have downregulated Notch signaling. Additional investigation is needed to determine the lineage-specific mechanism of CHD8’s pro-survival activity.

A growing body of literature shows that inhibition of chromatin-modifying proteins is a promising field of investigation and drug development. Inhibiting these factors may correct global transcriptional deregulation instigated by events such as Myc overexpression. Our work suggests CHD8 is a potential drug target in B cell malignancies provided toxicity in normal hematopoietic cells is not limiting. Our results and those of others indicate that inhibiting CHD8 would not be as effective in T cell malignancies driven by Notch signaling, and could be counter-productive in certain solid tumors. However, CHD8 inhibition could synergize with γ-secretase inhibitors in cells that have become resistant through epigenetic mechanisms. When compared to our knowledge of other chromatin-modifying proteins, our understanding of the function of CHD8 is relatively incomplete. Nonetheless, it is clear that CHD8 has critical roles in cell survival, and additional investigation should be conducted to better define its place within central signaling pathways.

## Supporting Information

S1 Fig CHD8 shRNAs cause similar levels of depletion of transduced cells in *in vitro* competition assays. Graph showing depletion of shRNA-expressing B-ALL cells *in vitro* over time. Shown are averages ± SEM of three independent experiments.
(PDF)

S2 Fig Deletions in *Chd8* sequence following transduction with sgRNAs targeting *Chd8*. A region surrounding the sgRNA binding site was PCR-amplified from genomic DNA extracted from the indicated clonal populations. PAM sequences are indicated in italics and stop codons are indicated in bold. Chd8 WT indicates the normal gene sequence.
(PDF)

S3 Fig CHD8 expression is retained in cells transduced with sgRNAs targeted to Renilla luciferase. Western blot showing CHD8 expression in B-ALL cells transduced with sgRNAs targeted to Renilla luciferase. Image was altered to juxtapose discontinuous lanes run on the same gel.
(PDF)

S4 Fig Locations in *Chd8* targeted by shRNAs. (PDF)

S5 Fig sh*Chd8*-mediated cell death is not rescued by treatment with the pan-caspase inhibitor ZVAD-fmk. BCR-Abl+ B-ALL cells transduced with TRMPVIR-shRen or TRMPVIR-sh*Chd8*-1 were plated in triplicate with doxycycline and ZVAD-fmk or DMSO vehicle control. The percentage of Venus^+^dsRed^+^ cells was assessed every two days by flow cytometry, and the fold change from time of plating to the end of the assay (eight days) was calculated. Shown are averages and SD of triplicate wells.
(PDF)

S6 Fig Caspase 3 cleavage is not observed upon induction of sh*Chd8*-1. A pure population of BCR-Abl+ B-ALL cells expressing TRMPVIR sh*Chd8*-1 was plated with or without doxycycline and collected at the indicated times. Protein lysates were generated and analyzed by western blot for the presence of cleaved (17 kDa) and pro-caspase 3 (53 kDa). Actin was used as a loading control. Image cropped to align discontinuous lanes from the same membrane.
(PDF)

S7 Fig ICN is overexpressed in KP lymphoma cells transduced with MIG-ICN. Western blot showing expression of ICN in KP lymphoma cells following transduction with *MIG-ICN*.
(PDF)

S1 Table Sequences of shRNAs targeting *Chd8*. (XLSX)

S2 Table Sequences of PCR and sequencing primers used in the study. (XLSX)

S3 Table Sequences used to construct sgRNAs used in the study. (XLSX)

S4 Table List of antibodies used in the study. (XLSX)

S5 Table Sequences of qPCR primers used in the study. (XLSX)

S6 Table Statistical analysis of *Chd8*-knockout survival study (Fig 2C). (XLSX)
