# Supplementary figures and images for: The Chromatin Regulator CHD8 Is a Context-Dependent Mediator of Cell Survival in Murine Hematopoietic Malignancies
Source: PLoS One. 2015 Nov 20;10(11):e0143275. doi: 10.1371/journal.pone.0143275 (PMC4654476; doi:10.1371/journal.pone.0143275)

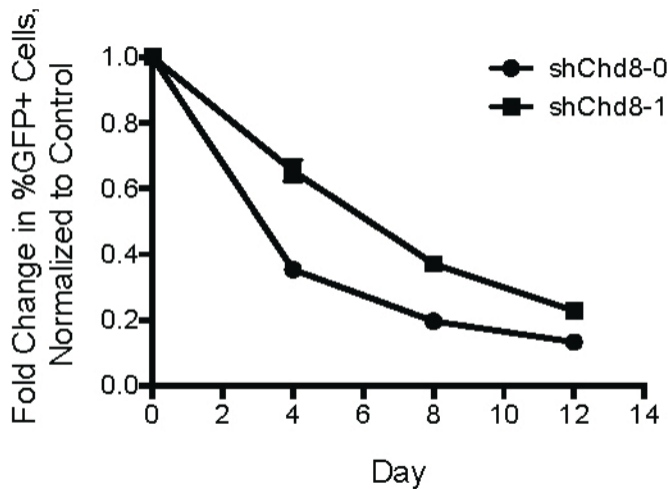

Supplement: S1 Fig — Graph showing depletion of shRNA-expressing B-ALL cells in vitro over time. Shown are averages ± SEM of three independent experiments. (PDF) [file pone.0143275.s001.pdf]

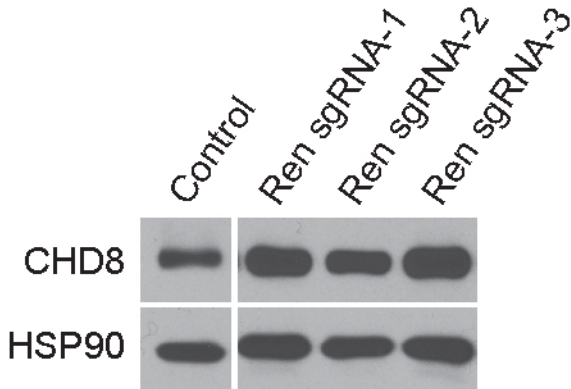

Supplement: S3 Fig — Western blot showing CHD8 expression in B-ALL cells transduced with sgRNAs targeted to Renilla luciferase. Image was altered to juxtapose discontinuous lanes run on the same gel. (PDF) [file pone.0143275.s003.pdf]

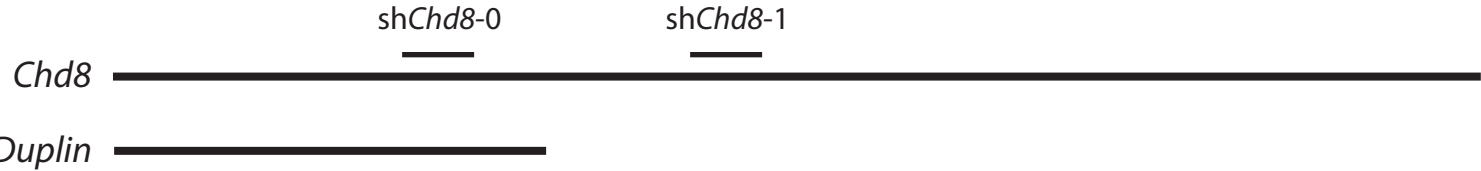

Supplement: S4 Fig — (PDF) [file pone.0143275.s004.pdf]

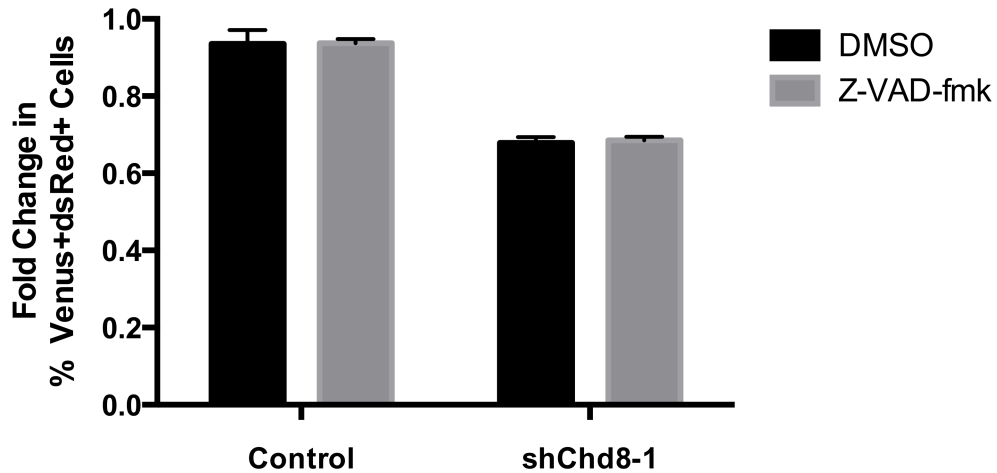

Supplement: S5 Fig — BCR-Abl+ B-ALL cells transduced with TRMPVIR-shRen or TRMPVIR-shChd8-1 were plated in triplicate with doxycycline and ZVAD-fmk or DMSO vehicle control. The percentage of Venus+dsRed+ cells was assessed every two days by flow cytometry, and the fold change from time of plating to the end of the assay (eight days) was calculated. Shown are averages and SD of triplicate wells. (PDF) [file pone.0143275.s005.pdf]

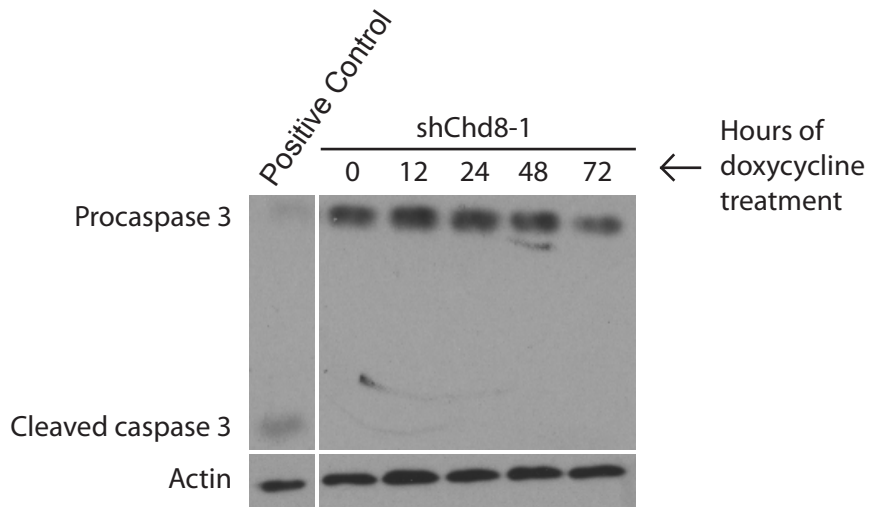

Supplement: S6 Fig — A pure population of BCR-Abl+ B-ALL cells expressing TRMPVIR shChd8-1 was plated with or without doxycycline and collected at the indicated times. Protein lysates were generated and analyzed by western blot for the presence of cleaved (17 kDa) and pro-caspase 3 (53 kDa). Actin was used as a loading control. Image cropped to align discontinuous lanes from the same membrane. (PDF) [file pone.0143275.s006.pdf]

Control  
MIG-ICN

ICN

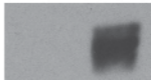

HSP90

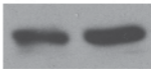

Supplement: S7 Fig — Western blot showing expression of ICN in KP lymphoma cells following transduction with MIG-ICN. (PDF) [file pone.0143275.s007.pdf]
