# Supplementary material for: The Chromatin Regulator CHD8 Is a Context-Dependent Mediator of Cell Survival in Murine Hematopoietic Malignancies
Source: PLoS One. 2015 Nov 20;10(11):e0143275. doi: 10.1371/journal.pone.0143275 (PMC4654476; doi:10.1371/journal.pone.0143275)
Supplement: S2 Fig — A region surrounding the sgRNA binding site was PCR-amplified from genomic DNA extracted from the indicated clonal populations. PAM sequences are indicated in italics and stop codons are indicated in bold. Chd8 WT indicates the normal gene sequence. (PDF) [file pone.0143275.s002.pdf]

Chd8 sgRNA-1: CTA CCA GGC -AGA **TAG**
Chd8 WT: 928 CTA *CCA* GGC AAG ATA G 943

Chd8 sgRNA-2: GGG AGT --- --- --- --- --- --- -TAC AGG GCA ACC AGC **TAG**
Chd8 WT: 922 GGG AGT CTA *CCA* GGC AAG ATA GTG TTA CAG GGC AAC CAG CTA G 964

Chd8 sgRNA-3: GTG T-- CA GGG CAA CCA .... **TAA** 1265
Chd8 WT: 943 GTG TTA CA*G GG*C AAC .... GTT AA 1265

Chd8 sgRNA-5: AAG ATA --- --- CAG GGC AAC
Chd8 WT: 937 AAG ATA GTG TTA CA*G GG*C AAC 957
